# Whither metabolic flux analysis in plants?

**DOI:** 10.1093/jxb/erab389

**Published:** 2021-08-25

**Authors:** Nicholas J Kruger, R George Ratcliffe

**Affiliations:** 1 Department of Plant Sciences, University of Oxford, South Parks Road, Oxford OX1 3RB, UK; 2 MPI of Molecular Plant Physiology, Germany

**Keywords:** Flux balance analysis, metabolic flux analysis, metabolic network, metabolic phenotype, non-stationary analysis, steady-state analysis

**Stable isotope labelling experiments provide many opportunities for probing metabolic pathways. One goal of such experiments is to define the metabolic phenotype of an organism in terms of the fluxes supported by the entire metabolic network. Metabolic flux analysis (MFA) is used routinely in prokaryotes, but its application to plants is more challenging. Here we examine the status of MFA in plants, highlighting difficulties that hinder wider exploitation of the technique. We conclude that simulation of network fluxes using constraints-based modelling offers a more versatile approach for exploring the capabilities of a network, and that MFA might be best used to probe the interesting features that simulation reveals.**

## Steady-state MFA

If an experimental system in a metabolic steady state can be labelled to an isotopic steady state, then so-called steady-state metabolic analysis can be used to construct a flux map for the principal pathways of central metabolism (Box 1). Although the fundamental assumptions that underpin the method are restrictive, steady-state ^13^C-MFA has made significant contributions to the analysis of network-wide metabolic responses, and the provision of energy and reducing power, in heterotrophic systems such as plant cell cultures and developing embryos (Kruger and Ratcliffe, 2015). The ability to resolve some fluxes at a subcellular level, providing quantitative data on parallel cytosolic, mitochondrial, and plastidic fluxes, is an important feature of the method. Technically the method continues to advance (Beyß *et al.*, 2021; Millard *et al.*, 2021), but the goal of a high-throughput implementation of the method in plants remains elusive.

**Box 1. Principles of isotope-assisted flux analysis in plants**

(A) Metabolic flux analysis (MFA) is based on a set of mass balance relationships describing flux through the network at metabolic steady state. The relationships are constrained by measurements of isotopomer abundances of metabolic intermediates and/or products at isotopic steady state following supply of a substrate labelled with a stable isotope. Least-squares regression analysis of this overdetermined problem is used to infer the combination of flux values in the network that best explains the observed redistribution of label. Heterotrophic networks are commonly analysed by steady-state MFA involving the supply of a partially ^13^C-labelled substrate. (B) In autotrophic networks involving the assimilation of a single carbon atom substrate, all metabolites become uniformly labelled at isotopic steady state, and analysis of such systems requires a transient ^13^C labelling experiment followed by non-stationary (INST) MFA. In principle, INST-MFA can also be used to interrogate heterotrophic networks, and this would be the appropriate strategy for investigating systems in which a metabolic steady state cannot be maintained for long enough to achieve an isotopic steady state after supply of a labelled substrate. (C) Both steady-state MFA and, to a lesser extent, INST-MFA are employed routinely for the analysis of non-compartmented metabolic networks in microbes. (D) The extension of MFA to distinguish between fluxes through equivalent reactions in different subcellular compartments of eukaryotic plant cells relies on the analysis of products that are synthesized in specific cellular locations. These products report on the labelling patterns of defined subcellular pools of network intermediates, and their usefulness depends on the extent to which the labelled intermediates exchange between different organelles. (E) Resolving the distribution of fluxes in different cell types in a complex tissue or organ similarly relies on measurement of labelling patterns of compounds that reflect the isotopomer profiles of network intermediates in individual cell types, and the ability to achieve this may be compromised by the nature and extent of exchange of metabolites between the different cell types.

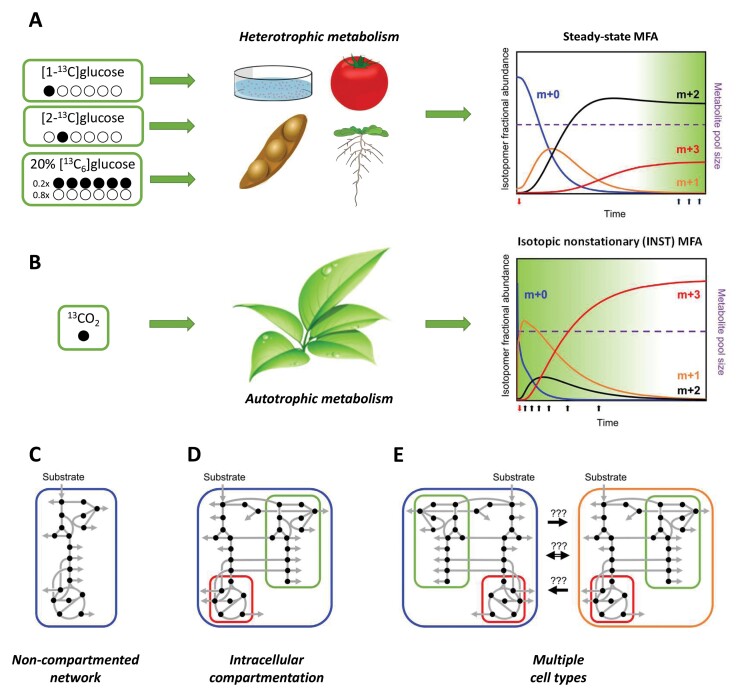

One limitation of steady-state ^13^C-MFA is that the provision of energy and reducing power can only be addressed indirectly because the interconversion of pairs of coenzymes does not involve a carbon transfer. This means that ^13^C-MFA cannot distinguish between the activity of NAD- and NADP-dependent enzymes, and this leads to uncertainty in deducing coenzyme budgets for the network. In principle, this problem might be solved using a deuterium labelling strategy developed in mammalian cells (Fan *et al.*, 2014), but when this method was used to assess the contribution of the oxidative pentose phosphate pathway to NADPH production in a heterotrophic *Arabidopsis thaliana* cell culture, the interpretation was confounded by the high rates of the exchange reactions catalysed by flavin-containing enzymes (Smith *et al.*, 2019).

A major unsolved limitation of ^13^C-MFA arises when the labelled system, for example a root tissue, is made up of multiple cell types with potentially different flux distributions in each cell type. In this situation, analysing the labelling of extracted metabolites does not necessarily produce a true average of the fluxes within the tissue, highlighting the need for cell-specific MFA techniques that would allow direct estimation of the fluxes in the different cell types within a tissue (Rossi *et al.*, 2017). A possible solution to this problem, similar in principle to the MFA approach used to deduce compartment-specific fluxes (Allen *et al.*, 2012), is to analyse the labelling of a protein that is expressed in a particular cell type, and to use that information to deduce the flux map for the corresponding cell (Rossi *et al.*, 2017). Although the principle has been demonstrated (Rossi *et al.*, 2017), this method has not yet been successfully implemented either using a protein with a restricted expression pattern, for example nitrogenase in a root nodule, or using a heterologous reporter protein expressed with a cell-specific promoter. It is unclear whether the practicality of the proposal will be undermined by exchange of metabolic precursors for protein synthesis between cell types, for example via plasmadesmata, or by an inability to deduce reliable fluxes from the labelling data in the absence of cell-specific information about the label inputs and outputs into the network. Ultimately the wider application of steady-state ^13^C-MFA to heterogeneous plant tissues depends on developing a practicable method for measuring cell type-specific fluxes.

## Non-stationary MFA

Photoautotrophic metabolism is a hallmark of leaves, but steady-state labelling with ^13^C-labelled carbon dioxide or bicarbonate leads to uniform labelling across the whole metabolic network, and thus provides no useful information about the fluxes responsible (Roscher *et al.*, 2000). Analysing labelling time-courses in a metabolic steady state using the technique known as non-stationary MFA (INST-MFA) (Wiechert and Nöh, 2013) is a potential solution to this problem, but so far there have been just two applications to leaves (Ma *et al.*, 2014; Xu *et al.*, 2021). These studies demonstrated that INST-MFA can provide a system-wide analysis of the metabolic fluxes in photoautotrophic metabolic networks, together with quantitative information on the pool sizes of metabolic intermediates. However, INST-MFA is even more experimentally demanding than steady-state MFA, since it requires measurements of the labelling patterns of metabolic intermediates at multiple time points, some of which must be made within minutes of the introduction of the label (Cheah and Young, 2018). These requirements, together with the complexity of the subsequent analysis, are an obstacle to the routine application of INST-MFA in plants and no doubt explain the paucity of applications to date.

A recent critique of the two published leaf studies highlights the challenges that need to be met if the approach is to be regarded as a source of reliable flux and pool size measurements for the central metabolic network (Wieloch, 2021). This analysis argued that investigator-led choices about the definition of the reaction network and the constraints applied to the model, together with uncertainties arising from the potential supply of unlabelled carbon to the network from internal recycling of carbohydrate stores, raised questions about the accuracy of the reported flux values and thus the reliability of the conclusions drawn from the data. The author also criticized some statistically weak claims about agreement with other observations and argued that validation of the deduced flux values and metabolite pool sizes should be a priority at this point in the development of the technique (Wieloch, 2021). Although validation of the outputs from a model would be reassuring, especially given the uncertainties inherent in defining the large-scale metabolic network models used in MFA, convenient and reliable methods do not generally exist for validating much of the information that emerges from INST-MFA.

In principle, INST-MFA can also be applied to heterotrophic plant tissues to allow flux analysis in systems that cannot be maintained in a metabolic steady state. For example, tissues responding to physiological perturbations are not amenable to steady-state MFA, because it may be difficult for the system to achieve an isotopic steady state that reflects the transient metabolic state at the point of measurement (Roscher *et al.*, 2000). There have been no INST-MFA studies to date of this kind, in which heterotrophic metabolism is changing during the labelling of the plant tissue. Even so, it must be recognized that the limitations that affect steady-state MFA, namely the indirect analysis of the coenzyme requirements and the confounding effects of cellular heterogeneity, would still exist for INST-MFA. In this situation, the current focus on photoautotrophic cells is understandable given that such tissues are not in any case amenable to steady-state MFA.

While MFA generally focuses on generating flux maps for extensive metabolic networks, it has recently been proposed that a variant of ^13^C-INST-MFA, ScalaFlux, could be used to extract information on individual steps or sections of the network (Millard *et al.*, 2020). ScalaFlux is a non-stationary technique that focuses on the propagation of isotopic label through a local subnetwork. It has many advantages, not least that it does not require an accurate description of the entire network, and while the subcellular compartmentation of plant metabolism may well complicate the application of ScalaFlux, the technique may offer a solution to more targeted analysis of flux changes.

## Simulation as an alternative to MFA

The stoichiometric structure of a metabolic network can be used as the basis for a multitude of modelling strategies (Suthers *et al.*, 2021), and the simulation of the fluxes through plant metabolic networks using constraints-based flux balance analysis (FBA) has emerged as a powerful alternative to measuring fluxes (Töpfer, 2021; Tong *et al.*, 2021). In outline, FBA enables the prediction of the flux distributions within a network that permit the conversion of substrates to products under specified conditions. This task requires the selection of one or more objectives that must be met by the network in achieving the formation of the products, for example minimizing the consumption of ATP, and it is also subject to known constraints on the network such as information on the input and output fluxes and the thermodynamic reversibility of reactions (Box 2).

**Box 2. Simulating fluxes by flux balance analysis**

(A) A metabolic network interconverts a set of intermediates (A, B, …) and thereby converts inputs (A, B) to outputs (E) at rates characterized by fluxes (*v*_1_, *v*_2_, …). The stoichiometric matrix provides a mathematical description of the network in which the rows represent metabolites and the columns represent reactions. Here, column 3 indicates that reaction 3 consumes equimolar quantities of A and B to produce intermediate C. (B) If the metabolic system is in a steady state, the concentration of every intermediate will be constant, and the product of the stoichiometric matrix and the flux vector will be zero. There may be many flux distributions that correspond to a metabolic steady state, and an optimal solution for a particular set of conditions can be found by using an optimization technique such as linear programming. Operationally it is necessary to apply a set of constraints to the network, such as substrate consumption rate, biomass composition. and known limits on specific fluxes, or more generally metabolomic, transcriptomic. or proteomic data, and to assume that the network operates to achieve a specific objective. The latter would typically be an efficiency objective, such as minimization of the sum of fluxes or maximization of growth. Note that in contrast to MFA, the computational problem is underdetermined, so the optimal solution is not unique. The extent to which the specified objective function can be satisfied by alternative flux distributions can be assessed using flux variability analysis. (C) The applications of flux balance analysis to plants can be greatly extended by setting up sets of models that provide a description of the system under different conditions and solving them as a single optimization problem. In this example, the system is described by five phases that are linked by transfers of cytosolic, mitochondrial, plastidic, and vacuolar metabolites. More generally, the different models might represent different tissues in a whole plant, or different cell types in a tissue, or different time points across a developmental time scale. In the simplest application, the impact of the diel cycle on leaf metabolism can be modelled by setting up light and dark models, with linking metabolite fluxes, and solving the two models simultaneously.

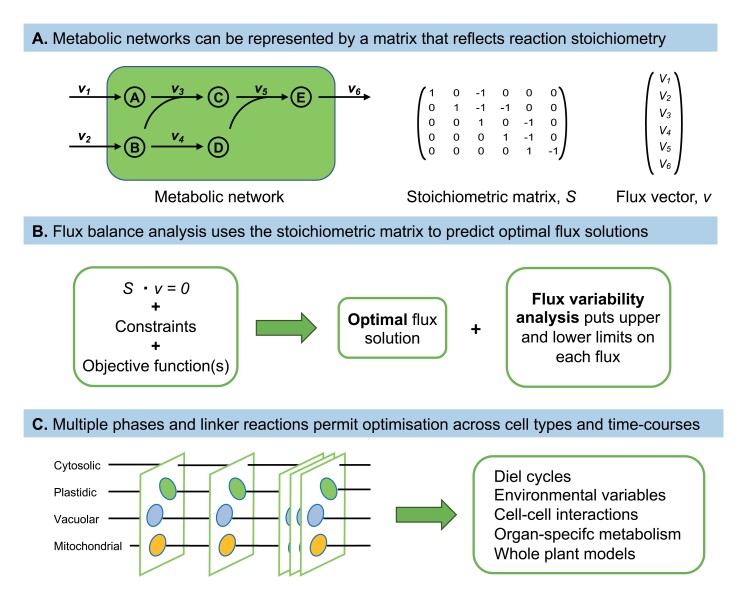

The selection of the constraints is very important in determining the validity and usefulness of the flux distributions predicted by FBA, but there has been more than a decade of experience in using this technique in plants, and the technique is now well established as a method for exploring the metabolic capacity of the plant metabolic network. Recent applications of the technique have included an analysis of leaf energy balance in the light (Shameer *et al.*, 2019), which highlighted a role for the export of reducing equivalents from the chloroplast to support photorespiration, and the use of a time-resolved diel model to analyse the trade-off between leaf productivity and water saving under different environmental conditions (Töpfer *et al.*, 2020).

Given the difficulties that have arisen in the application of empirical MFA to plants, there is a strong case for exploiting the power of stoichiometric modelling to identify situations in which the investment in time and effort for MFA would be justified. For example, FBA models can be used routinely to predict the consequences of metabolic engineering (King *et al.*, 2015), and verifying those predictions experimentally, using targeted approaches such as ScalaFlux, may be the future for ^13^C-MFA in plants.
